# Narrative Review of Human-Centered Design in Public Health Interventions in Low- and Middle-Income Countries: Recommendations for Practice, Research, and Reporting

**DOI:** 10.9745/GHSP-D-24-00164

**Published:** 2025-08-14

**Authors:** Bee-Ah Kang, Manvi Poddar, Aditi Luitel, Rajiv N. Rimal, Biruk Melaku, Danielle Piccinini Black

**Affiliations:** a Department of Health, Behavior & Society, Johns Hopkins Bloomberg School of Public Health, Baltimore, MD, USA.; b Department of International Health, Johns Hopkins Bloomberg School of Public Health, Baltimore, MD, USA.; c Albert Einstein College of Medicine, New York, NY, USA.; d Johns Hopkins Center for Communication Programs, Addis Ababa, Ethiopia.; e Johns Hopkins Center for Communication Programs, Baltimore, MD, USA.; f Johns Hopkins Carey Business School, Baltimore, MD, USA.

## Abstract

The application of human-centered design (HCD) is growing in global health, given its potential to generate innovative solutions to entrenched health problems by prioritizing human perspectives, needs, and desires. To address gaps in consolidated evidence on prior programs, we conducted a review of studies that applied a comprehensive HCD approach in low- and middle-income countries. A total of 535 articles were initially identified. Based on the inclusion and exclusion criteria, 22 articles were included. Most studies were conducted in sub-Saharan Africa and used qualitative or mixed methods throughout the HCD work. In the “discover and define” phase, user personas, journey maps, and interviews were commonly used to empathize with end users and key stakeholders. Studies used various strategies in the “ideation” phase, including idea generation based on feasibility and resource constraints. In the “testing” phase, low-fidelity prototypes were tested to obtain feedback from end users and stakeholders, enabling quick and cost-effective refinements. Prototype iterations occurred twice in most studies, but information about when iterations ceased was limited. Evaluations of design outcomes and health impacts were lacking. Studies cited multidisciplinary approaches, flexible methodology, and a sense of ownership among users and communities as strengths of HCD. Contrastingly, challenges in consistent participant engagement and limited scientific rigor were reported as weaknesses. Elements that enhanced program reporting included clear descriptions of HCD as cyclical, stakeholder maps (empathy tools), visual materials on design activities and prototypes, and transparency in failures. We recommend strengthening capacity among those applying HCD to optimize the effectiveness of the approach for global health. Although HCD is not inherently intended to serve as a rigorous research method, data triangulation and proper evaluations may ensure its usability as evidence in health research when appropriate. Also, a thorough reporting of design phases and providing detailed rationale behind design decisions can advance future HCD literature.

## Abstract (precis)

Although human-centered design is increasingly used in global health, its comprehensive application in health programs remains underexplored. Our narrative review demonstrates design tools and strategies, research methods, and reporting patterns across the included studies to provide practical recommendations.

## Teaser

**Key Findings:**

- Human-centered design (HCD) methods and strategies were distinct by design phase. In the “discover and define” phase, studies used multiple design tools and research methods to fully empathize with end users. In the “ideation” phase, studies allowed innovative ideas to emerge, followed by prioritizing ideas based on feasibility and resource constraints. In the “testing” phase, rapid testing enabled quick and cost-effective refinements, with 2 rounds of iterations being the most common.
- Monitoring and evaluations of design outcomes and health impacts were lacking. A lack of monitoring and evaluation efforts and scientific rigor has been cited as a weakness of HCD.
- Reporting patterns greatly varied. Descriptions of the cyclical nature of HCD, stakeholder maps, and visual materials on design activities and prototypes enhanced an understanding of the programs.

**Key Implications:**

- Program implementers and donors should invest in the capacity-building of health practitioners using HCD to foster their skills in engaging underresourced groups and stakeholders, as well as in implementing and scaling designed solutions.
- Design practitioners should be trained to collect data from diverse sources for deeper empathy with end users and stakeholders, contributing to the scientific rigor of an HCD-based program.

## INTRODUCTION

Human-centered design (HCD) is an iterative approach to problem-solving that prioritizes the needs, desires, and behaviors of the people central to the problem and for whom solutions are being designed.^1^ It has been used as a strategy for designing and implementing public health interventions by engaging stakeholders and communities in the process of planning, research, design, and delivery.^2^ HCD affords unique advantages in solving complex health problems that are often intertwined with multiple layers of systemic challenges, competing priorities, and cultural diversity across communities. This article synthesizes and illustrates patterns of design practices, research, and reporting derived from evidence on HCD-based global health interventions.

Across health practices and research, diverse empathy-centric problem-solving processes have been used with various associated terms. Design thinking highlights the feasibility and desirability of a solution that matches people’s needs and values,^3^ while user-centered design places a stronger emphasis on employing end users as informants of a product or service.^4^ Social design is a methodology for changing human perceptions and beliefs^5^ to achieve equitable solutions.^6^ Despite the subtle differences conveyed by these terms, there may not be full consensus on their precise meanings within the design world. Nevertheless, they have been used interchangeably with HCD in health interventions, attending to human experiences and values in finding solutions.

Health organizations and practitioners have conceptualized HCD in a myriad of ways. Some models follow the 5-step layout—empathize, define, ideate, prototype, and test—described as design thinking.^7^ The Double Diamond^8^ and Harvard^9^ models encompass 4 distinctive steps. IDEO.org presents a simpler framework involving inspiration, ideation, and implementation.^1^ Synthesizing diverse processes, Mishra and Sandhu^10^ found 3 common design phases. Adapting these phases, we theorized that HCD is a holistic framework and includes (1) identifying problems by empathizing with people experiencing challenges; (2) ideating solutions and developing prototypes; and (3) testing, refining, and iterating on prototypes. Table 1 depicts these phases along with key design terms and methods identified from the literature.

**TABLE 1.** Phases of the Human-Centered Design Process Applied to Public Health Practices

	**Phase 1: Identifying and Analyzing Problems**	**Phase 2: Ideating Solutions and Developing Prototypes**	**Phase 3: Testing, Refining, and Iterating on Prototypes**
Definition	Understanding perspectives, challenges, needs, and desires of people through empathizing, identifying key insights, and creating a roadmap to innovative solutions	Generating solution ideas based on insights gained from empathy-driven research, building rough prototypes, and gathering feedback from key stakeholders	Implementing, testing, and iteratively refining prototypes
Related design terms	Empathize, define, discover, inspiration, inquire	Ideate, imagine, develop, prototype	Test, deliver, implementation, try, refine
Common design methods	User personas, video ethnography, in-depth interviews, journey maps, brainstorms, observations, mind wash, stakeholder mapping, user stories, day in the life	Brainstorm, mind maps, co-design workshops, role-play, “how might we…?” questions, list of attributes, brainwriting, storyboarding	Journey maps, ranking, paper prototypes, storyboarding, pre/post survey, pilot tests, post-study usability questionnaire

While the adoption of HCD in health intervention is relatively new, public health has a history of using person-centered research methods, such as participatory action research and trials of improved practices, which have helped lay the foundation for HCD in the field. It aligns well with the research shift in low- and middle-income countries (LMICs), which emphasizes strengthening local capacity and cultivating community-driven solutions.^6^ HCD grants ownership to those for whom the program is designed, curbing biases permeating into intervention development.^11^ Moreover, innovative and long-lasting solutions are more likely to be engendered, compared with traditional research that is often linear in testing innovations based on expert knowledge.^10^

The time trend of documentation from the U.S. Agency for International Development (USAID) indicates that the use of HCD in global health has increased exponentially since 2016.^10^ While evidence is still emerging, many programs in LMICs have applied HCD in health commodities,^12^^,^^13^ health education and counseling services,^14^^–^^16^ and health care and information systems^17^^,^^18^ based on empathy-centric and multidisciplinary principles. For example, a study conducted in Kenya used HCD to enhance its HIV treatment interventions based on end user insights.^19^ Similarly, the Billion Girls CoLab generated girl-led health solutions in Kenya, shifting power dynamics between girls and adult stakeholders.^20^^,^^21^

Meanwhile, HCD in LMIC settings has presented its own unique challenges. One major concern is the scarcity of financial resources and time constraints,^22^ which often results in lack of HCD-trained personnel and robustness of solutions. Additionally, cultural factors play a critical role in shaping stakeholder engagement; varying beliefs and practices can obstruct the integration of HCD principles within local contexts.^23^ Moreover, fragmented health systems may complicate the implementation of HCD approaches.^24^

Therefore, it is essential to gain a holistic understanding of how prior interventions have employed design principles in global health. A 2021 supplement issue in *Global Health: Science and Practice* was dedicated to guiding future practice by showcasing several HCD programs. A review of evidence is additionally needed to facilitate summative and comparative interpretation. Several literature reviews and case studies captured specific health topics, types of design outputs, or large-scale projects.^25^^–^^27^ Other reviews combined cases of LMICs with those of high-income countries, limiting our understanding of strategies for navigating programmatic and systemic challenges distinctively salient in resource-limited environments.^28^^–^^30^ The gap also stems from health programs often relying on certain elements of HCD rather than adopting a comprehensive process. While HCD as a “spark” or an “ingredient” in a project can be effective, we focused on projects that adopted an “end-to-end” process,^31^ as we believe those maximize empathy and drive the best innovations for users.

It is essential to gain a holistic understanding of how prior interventions have employed design principles in global health.

This narrative review aims to elucidate how HCD is employed and documented in the literature and offer recommendations for future research, practice, and reporting. Specifically, we seek to (1) describe programs that have adopted a comprehensive HCD approach; (2) critically analyze methods, strategies, and lessons learned across design phases; (3) examine monitoring and evaluation of programs to understand their impact; (4) consolidate programs’ strengths and limitations; and (5) explore reporting patterns in HCD literature.

## METHODS

We conducted a narrative literature review,^32^ chosen for its suitability in synthesizing information from diverse sources, given the practice-based and multidisciplinary nature of HCD programs. The inclusion criteria were studies that (1) described public health interventions, (2) applied a comprehensive design process (i.e., including all 3 phases in Table 1), (3) were conducted in LMICs per World Bank’s 2022 categorization,^33^ and (4) were published from 2010 through the search date of April 14, 2022. The time period was determined based on our preliminary literature search showing little to no HCD work in LMICs published before 2010. Articles were excluded if studies (1) did not incorporate a comprehensive design process or describe it in detail, (2) did not involve public health interventions (e.g., clinical interventions), (3) were conducted in upper-middle or high-income countries, or (4) were not original research (e.g., protocol articles) or articles not related to HCD. Gray literature was not included, given the absence of a peer-reviewed process to determine quality.

The literature search was conducted using PubMed and Google Scholar following an exploratory search across other databases (i.e., Scopus and Embase), which yielded limited results on HCD and other design-based approaches or primarily focused on biomedical and clinical research rather than public health interventions. Search domains and terms were developed in January 2022 by 2 researchers and were additionally reviewed by 2 additional team members. The main search domains were “human-centered design,” “public health intervention,” and “LMIC”; the domains and specific search terms under each domain were finalized upon agreement among the team of 4 in April 2022. We initially identified 442 articles on PubMed using the finalized strategy. Given HCD’s relatively nascent introduction to the scholarship, the team agreed to modify the strategy to include broader terms, such as “design” and “design thinking,” which led to the identification of 70 additional articles after removing duplicates. This change in our search strategy captured a wider range of studies, including some irrelevant ones, and inadvertently introduced bias. We mitigated these issues by using a 2-stage screening process for the retrieved articles, applying rigorous inclusion/exclusion criteria, and documenting the search process to ensure transparency.

The search on Google Scholar identified 2 additional articles after removing duplicates. Title and abstract screening of the 514 articles identified on PubMed and Google Scholar was conducted by 1 researcher. We excluded 471 articles as they did not meet inclusion criteria because they did not use or document a comprehensive HCD approach, were not related to health interventions, or were conducted in high-income countries. This led to 43 articles being entered into full-text screening. To complement our database search, we conducted a targeted search. References of the 3 literature review articles identified from the databases, along with scholarly journals that frequently emerged from the databases, were additionally reviewed. From these processes, 11 and 10 articles were identified from the reference and journal search, respectively. This subsequently led to an additional 17 articles being included in the full-text screening phase.

Full-text screening of 60 articles was conducted from September to October 2022 by 2 researchers. Any conflicts raised were resolved by a third researcher. The study team regularly convened to iteratively review screening results and reach consensus on final studies. We excluded 32 articles because they were not related to HCD or inadequately used or described the comprehensive process of the HCD approach. We also excluded 6 articles as they were not original research or reports on HCD implementation. In total 22 articles were included. The flowchart is presented in the Figure.

**FIGURE:**
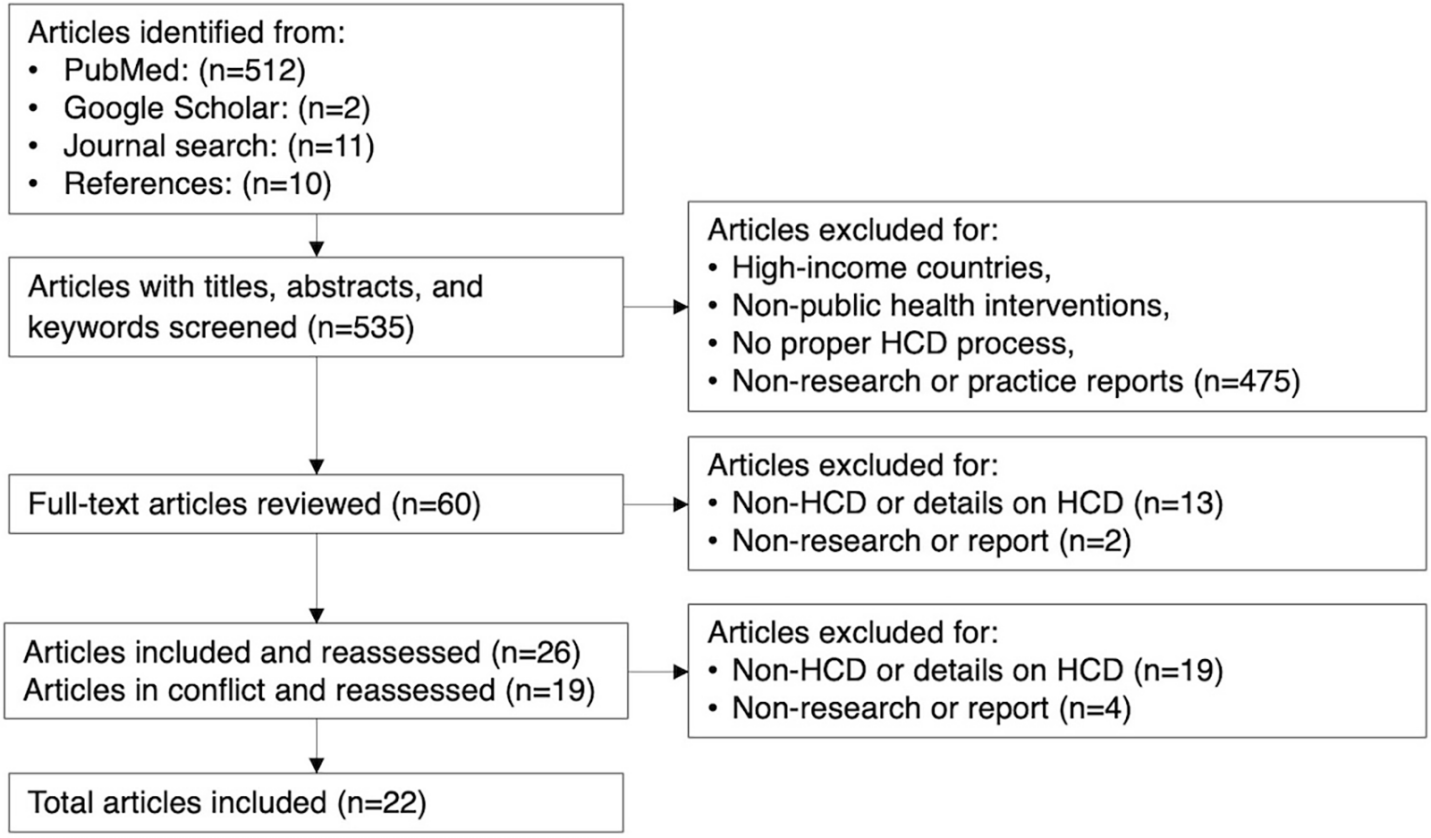
Flowchart of Studies Included in Review on Human-Centered Design Approach Applied in Low- and Middle-Income Countries

A data extraction template (codebook) was developed and finalized by 2 researchers. From December 2022 to March 2023, data extraction and analysis were conducted by 1 researcher and additionally reviewed by 2 team members. Themes and categories for data extraction were identified. Study characteristics, including country setting, health issue, target populations, study design, and methods, were recorded during the extraction procedure following the template. Information about studies’ use of HCD frameworks, ways in which HCD elements were used, design challenges and solutions, HCD outputs and outcomes, health impacts, lessons learned, and HCD strengths and limitations were collected. Data extraction and analysis were inductively conducted, given the heterogeneity of study designs and methods. Each article was also carefully reviewed by a second reviewer to ensure the quality of the initial data and to extract additional information according to the identified categories as needed. The procedure was performed using Excel.

## RESULTS

### Study Characteristics

A total of 22 studies were included in this review. Of those, 18 (81.8%) were conducted in Africa, 3 (13.6%) in Asia, and 1 (4.5%) in South America (Table 2). Study populations included adolescents, pregnant women, health providers, and individuals with certain health conditions. All studies extensively engaged relevant stakeholders across different stages of HCD. Health areas included infectious diseases (N=10) (e.g., HIV, TB, influenza pandemics), noncommunicable diseases (N=12) (e.g., hypertension, palliative care, cancer), and mental health. Thirteen (59.1%) studies were guided by or adapted existing HCD frameworks.

**TABLE 2.** Studies on Public Health Interventions That Used a Human-Centered Design Approach

**Field of Practice**	**Study**	**Setting**	**Population**	**Health Area**	**Study Method**
Social and behavioral change through community engagement	Fakoya et al., 2022^35^	Nigeria, Ethiopia, Tanzania	Girls aged 15–19 years	Sexual and reproductive health (contraception use)	Qualitative
	Person et al., 2016^36^	Zanzibar	School children	Urogenital schistosomiasis	Qualitative
	Kim et al., 2019^37^	Ghana	Middle-class citizens	Malaria prevention	Mixed
	Isler et al., 2019^38^	Burkina Faso	Mothers, pregnant or breastfeeding (aged 18 years or older)	Maternal nutrition, mHealth	Qualitative
	Ippoliti et al., 2021^39^	Rwanda	Adolescents and young adults	Family planning and reproductive health	Mixed
	Bruns, 2021^40^	South Africa	Men living with HIV	HIV	Mixed
Data monitoring and surveillance systems for disease outbreaks	Durski et al., 2020^42^	Guinea, Liberia, and Sierra Leone	Data collection officers, epidemiologists, IT staff, data managers, and laboratory personnel	Data collection and reporting for Ebola outbreak	Qualitative
	Tijani et al., 2021^43^	Nigeria	General public and patients affected by COVID-19	Health data accuracy and management for COVID-19	Mixed
	Shaikh et al., 2022^44^	India	Clinicians responsible for data entry, public health officials managing potential disease outbreaks	mHealth and disease outbreaks	Qualitative
Reframing health service delivery	Pillsbury et al., 2021^47^	Kenya	Patients referred for hypertension and clinicians	Hypertension	Qualitative
	Leung et al., 2020^48^	Kenya	Patients with NCDs like hypertension and diabetes	NCDs	Qualitative
	Arrivillaga et al., 2020^49^	Colombia	Women aged 25–65 years	Cervical cancer prevention	Mixed
	Yadav et al., 2021^50^	Nepal	People with multimorbid COPD	COPD	Mixed
	van der Westhuizen et al., 2020^51^	South Africa	The elderly	Increasing medication compliance	Qualitative
	Ramaswamy et al., 2018^52^	India	Citizens	Mental health	Mixed
mHealth interventions with end user engagement	Morse et al., 2021^55^	Tanzania	Cancer patients, palliative care specialists, and local health workers	Palliative care for cancer patients	Qualitative
	Farao et al., 2020^56^	South Africa	Health workers	Tuberculin skin test for TB infection	Mixed
	Ahonkhai et al., 2021^57^	Nigeria	Adolescents and young adults	HIV	Qualitative
System strengthening in multi-layered health sectors	Andersson et al., 2021^54^	Kenya	Seminomadic and nomadic people	Supply chain	Qualitative
	Ben-Zeev, 2021^59^	Ghana	Patients affected by serious mental illnesses	Mental illnesses	Qualitative
	Kanagat et al., 2021^59^	Nigeria and Democratic Republic of the Congo	Donor organizations and recipient countries	Technical assistance in maternal and child health	Qualitative
	Catalani et al., 2014^61^	Kenya	Clinicians	TB and HIV	Mixed

Abbreviations: COPD, chronic obstructive pulmonary disease; NCD, noncommunicable disease.

We identified the following 5 common fields of global health practice that leveraged a comprehensive HCD approach.

In the included studies, 5 common fields were identified that leveraged an HCD approach.

1. Social and behavior change aims to foster positive practices among individuals or communities to reduce inequity.^34^ Six studies^35^^–^^40^ (27.3%) used HCD to develop services or products to promote health behaviors.
2. Data monitoring and surveillance systems contribute to the continuous collection, analysis, and interpretation of data.^41^ Three studies^42^^,^^44^ (13.6%) used HCD to enhance data systems that aimed to monitor disease outbreaks.
3. Health service delivery is key to health systems, ensuring coverage for the intended population and providing quality care.^45^^,^^46^ Six studies^47^^–^^52^ (27.3%) focused on providing individuals with enhanced health services.
4. Digital health interventions refer to technology-based interventions for clients, health providers, and data services.^53^ Although some studies^38^^,^^39^^,^^54^ in other fields also featured digital components, 3 studies^55^^–^^57^ (13.6%) highlighted the nexus of user and functional requirements.
5. Health systems strengthening focuses on improving functions of service delivery, health workforce, information systems, and leadership.^58^ Four studies^54^^,^^59^^–^^61^ (18.2%) developed interventions to strengthen existing health systems in various sectors.

### Methods, Strategies, and Issues Emerged From Human-Centered Design Phases

We have organized our analysis results into 3 design phases applied to all included studies. We described common HCD methods (i.e., design tools), research methods, issues, and strategies unique to each design phase, presenting illustrative examples from a few studies (Table 3, with a comprehensive list in the Supplement). Box 1 summarizes the lessons learned in each design phase reported by the studies, and Box 2 summarizes the common HCD methods, principles, and strategies across design phases.

**TABLE 3.** Examples of Studies That Used HCD Approaches Across Design Phases

**Study**	**Phase 1: Identifying and Analyzing Problems** ^a^	**Phase 2: Ideating Solutions and Developing Prototypes** ^a^	**Phase 3: Testing, Refining, and Iterating on Prototypes**	**HCD Framework** ^a^
Fakoya et al.^35^	Inquiry involved activities such as observations, photo narratives, card-sorting, and in-depth interviews. Youth designers were engaged in this process, and market analysis was conducted to understand the benefits and drawbacks of delivery methods of a youth-friendly sexual and reproductive program. Evidence was systematically reviewed.	The data were used to focus on actionable insights to better connect girls to adolescent and sexual reproductive health services. All prototypes were tested for desirability, feasibility, and viability.	A final design was selected based on feedback from girls, their social networks, and health systems stakeholders. The intervention was tested in an adaptive implementation approach to assess the continuous program improvement.	Five instrumental strategies for integrated HCD and community-based participatory research^18^
Durski et al.^42^	Empathy was evoked to gain situational awareness and understand the needs of each stakeholder and end user group regarding an Ebola data system. Their needs were defined.	Ideas were generated using activities such as brainstorming. A prototype of the data collection and reporting system for Ebola was developed with the help of IT system engineers and computer programmers.	The prototype was tested internally by the IT team and rolled out in the in-country contexts.	Design thinking model by Hasso-Plattner Institute of Design at Stanford
Arrivillaga et al.^49^	Observations and interviews were conducted to identify challenges with cervical cancer prevention. A stakeholder workshop was held to develop user personas and journey maps using “how might we…?” questions as a part of the needs synthesis process.	Fifteen workshops were conducted as part of the ideation and co-design process. Activities included brainstorming, blue slips, list of attributes, and brainwriting. Participants voted on the ideas that they would like to see built during the prototyping phase of cervical cancer prevention interventions.	Viability of prototypes was evaluated along with their usability. After refining the prototypes, their usability was tested in health centers.	N/A
Morse et al.^55^	The study was divided into 5 phases that were guided by HCD principles. Defining a set of 3 app design requirements was followed by developing user personas to define user requirements. User stories were also created.	A draft data architecture was developed along with display pages and input forms to create the prototype for a palliative care app for cancer patients. A user experience expert then reviewed the prototype.	The prototype was then tested for usability. Challenges with usability were recorded. A final prototype was tested in a pilot.	3-phase methodology^19^
Ben-Zeev et al.^59^	Interviews were conducted to gather information on current practices, needs, readiness to change current practices, and assess comfort in using technology among traditional healers.	A co-design session was held to ideate and assess the feasibility of the design directions of a toolkit for traditional healers. Mood board prototypes were used along with projected slides detailing different content delivery modalities. Content development was guided by stress-vulnerability model and social rank theory. The prototype development included details on information architecture, branding, and animated video and text content.	Usability testing was conducted to assess the perceived usefulness and usability of the intervention for traditional healers who provide care to people with mental health illnesses.	N/A

Abbreviations: HCD, human-centered design, IT, information technology.

^a^ N/A indicates there was no particular framework or process stated.

BOX 1 Lessons Learned From Different Design Phases, as Illustrated by Studies

Phase 1

- To optimize empathy with populations experiencing disadvantages, design teams should be trained to ensure ethical and safe participation of the users throughout all human-centered design (HCD) activities.^39^
- It is worthwhile to broaden the definition of the term “user” to evoke empathy not just with the primary target groups but also with secondary stakeholders whose perspectives are critical in developing and implementing an intervention.^35^
- Conducting diverse research methods and gathering data from various sources enrich the empathy process, offering profound insights into user needs and problem-solving strategies.^36^
- There is a need for transparency in how input from end users and key stakeholders are incorporated into design team discussions and decisions.^47^

Phase 2

- Facilitators of ideation activities must be skilled to elicit thorough information equally from all users and stakeholders.^36^
- Inviting diverse local stakeholders, including those who share insights into feasibility issues, makes the implementation of ideation process more successful.^50^
- Ensuring diverse feedback from participants in the ideation phase helps foster idea development.^56^ Designers must be well versed in synthesizing and validating ideas.^35^
- New ideas that may challenge the convention must be appreciated. Embracing possibilities pushes designers toward creative solutions to intractable problems.^36^

Phase 3

- Iterative testing and refinement of prototypes guided by scientific evidence may alleviate tensions stemming from prototypes that challenge existing knowledge.^36^
- Monitoring and impact evaluation must be integral components of the design process.^44^
- Transparency in selecting design discussion topics, prioritizing solution ideas, and incorporating feedback from external study investigators is needed.^35^^,^^47^
- Follow-up studies are needed to explore how interventions are implemented in full scale and scaled-up in larger communities.^40^^,^^49^^,^^50^^,^^54^^,^^59^

BOX 2 Summary of Common Human-Centered Design Methods, Principles, and Strategies Across Design Phases

- Strong emphasis on evoking empathy with end users to identify design challenges, relying on data triangulation of design tools and traditional research methods, such as in-depth interviews.
- Comprehensive engagement of diverse stakeholders to better grasp end users’ needs, gather relevant contextual information, and achieve mutual understanding among key partners.
- Ideation focusing on exploring novel ideas that were previously overlooked or not reflected in other existing interventions while also considering key programmatic factors and specific features of intended interventions.
- Rapid testing of low-fidelity prototypes in “rough” and creative forms, enabling end users and stakeholders to provide honest feedback through various testing methods tailored to the type of prototype.
- Iterative testing and refinement alleviating tensions between new prototypes and existing knowledge and addressing conflicts between user desirability and feasibility concerns.

### Phase 1: Identifying and Analyzing Problems

As a preliminary step, all studies placed a strong emphasis on evoking empathy for people with lived/living experiences and identifying design challenges (Table 3). Design tools used at this stage included user personas, actionable problem statements, insight statements, and journey maps, and studies commonly employed multiple methods within this phase. For example, a study^35^ conducted in Nigeria, Ethiopia, and Tanzania used observations, photo narratives, and card-sorting with teams of youth and adults to maximize empathy and develop a sexual and reproductive health intervention for adolescent girls. Another study redefined “users” at the outset by cultivating empathy not just with the priority population but also with service providers and other supply-side stakeholders.^35^ The “empathy stage” often required additional strategies to ensure the safe and ethical engagement of users, given the unique set of vulnerabilities and power differentials among them. For example, the engagement and proper training of local youth/peer designers ensured the protection and sustained participation of underserved adolescent users.^39^

In addition to HCD methods, studies simultaneously integrated varied traditional research methods to further comprehend user needs and context. Qualitative methods were predominantly used, given their inherent merit in obtaining experiential and contextual insights from target groups. One study^36^ from Zanzibar conducted in-depth interviews, focus group discussions (FGDs), and observations as tools for empathy to understand perceptions, attitudes, and social norms around schistosomiasis to develop disease control interventions in the community. Thorough data analysis and strategic data collection enabled the design team to overcome challenges posed by initially uninformative or socially acceptable responses.^36^

Also, all studies extensively engaged diverse stakeholders to grasp end users’ needs, understand relevant contextual information, and achieve mutual understanding among key partners. While stakeholder engagement was integral to all design phases, convening a team of key stakeholders to create a sense of commitment and ownership was particularly highlighted in this earlier phase. A study^42^ that focused on building the Ebola data system involved data collection officers, epidemiologists, laboratory personnel, technical experts, and senior leadership in this manner. However, the engagement of stakeholders from interdisciplinary fields may present challenges due to conflicting opinions. The need for power-sharing was emphasized to ensure end users’ voices are represented in major design decisions.^47^

All studies extensively engaged diverse stakeholders to grasp end users’ needs, understand relevant contextual information, and achieve mutual understanding among key partners.

### Phase 2: Ideating Solutions and Developing Prototypes

Studies ideated on potential solutions based on insights gathered in Phase 1 to develop low-fidelity prototypes. Common HCD tools used to ideate solutions and build prototypes included storyboarding, brainstorming, “how might we…?” questions, and co-design workshops with stakeholders and end users. Embracing possibilities and new ideas were celebrated in this phase, as they pushed designers towards creative solutions.

Ideation occurred using various techniques. First, several studies focused on generating novel ideas that were previously overlooked or not reflected in other interventions. Aiming to redesign supply chains in Northern Kenya, a study^54^ leveraged research guides and user mapping to obtain information from key health personnel as insights for design. Second, some studies ideated solutions considering key programmatic factors or specific attributes of intended interventions. One study^42^ collected ideas primarily centering around the features and functions of infectious disease data systems while considering time and resource constraints in system development based on input from key stakeholders.^50^ Lastly, ideation often occurred following a procedural framework. A study^50^ from Nepal adopted a process of divergent thinking (i.e., brainstorming) followed by convergent thinking (i.e., refining and integrating) to synthesize ideas into an applicable concept. Throughout the ideation process, the role of designers in eliciting diverse and genuine insights as well as synthesizing information to produce tangible representations was largely emphasized.^35^^,^^36^

Prototypes were then developed by materializing ideated information. The use of research methods was generally scarce, but a study^37^ in Ghana underlined that methods like FGDs can be facilitated throughout the entire project to follow unexpected and interesting threads in participants’ conversations and confirm insights. Most technology-based prototypes were developed incorporating both functional and user requirements. For example, a data system development was guided by system capabilities, including data ownership, security, and network connection, while concurrently accommodating user needs.^42^

### Phase 3: Testing, Refining, and Iterating on Prototypes

The process of translating design challenges into solutions culminated during this phase (Table 4). Studies used various methods to test and refine prototypes. For example, community urinals were tested through observations of community use and maintenance,^36^ whereas service products were tested using workshop sessions and monitoring records.^47^ A feature of testing that is uniform across all studies was the engagement of multidisciplinary teams. A study tested 73 prototypes of a sexual and reproductive health program, engaging end users, government stakeholders, and health workers, as they offered insights into the desirability, feasibility, and viability of the solutions.^35^ Given that multiple ideas are narrowed down to finite solutions, ensuring transparency in prioritizing solution ideas was cited as a vital design principle.^35^^ ,^^47^

**TABLE 4.** Translation of Design Challenges Into Solutions and Evaluation Outcomes, by Field of Practice

**Study**	**Design Challenges**	**Design Solutions or Interventions**	**Monitoring and Evaluation** ^a^
Social and behavioral change through community engagement			
Fakoya et al.^35^	Limited understanding of social and cultural influences on adolescents and young adults specific to each country. Lack of awareness about contraception among newly married couples.	The interventions offer girls elements, such as life goals planning and financial skills, as part of the strategy to connect girls with youth-friendly contraceptive counseling and on-demand method provision.	N/A
Person et al. ^36^	Lack of awareness about how schistosomiasis is transmitted. Limited access to urinals, safe play activities, and laundry spaces.	Classroom-based education to increase knowledge about positive prevention behaviors among children; Kichocho safe play day events to highlight and educate safe play alternatives to risky, contaminated water play; installation of urinals close to open water bodies; washing platforms to discourage washing clothes in freshwater bodies.	The trained teachers had trained 678 of 761 registered in study areas. The 678 teachers conducted at least 1 educational session with 27,819 children of 30,034 possible students registered in the Unguja and Pemba study schools.
Kim et al.^37^	Unsatisfactory experience of using long-lasting insecticidal nets and irregular usage.	Prototypes built focused on convenience while hanging the net, more attractive designs, and zipper than allows easier entry and exit while maintaining a sealed, mosquito-free space.	N/A
Isler et al.^38^	Lack of contextually appropriate mHealth videos that are local to the context of Burkina Faso.	Adapted videos and illustrations to reflect local communities, homegrown/local vegetation, households as they appear in Burkina Faso, and local languages.	N/A
Ippoliti et al.^39^	Limited access and low quality of family planning and reproductive health services. Provider bias and judgement. Lack of quality materials for teachers to deliver information on comprehensive sex education in schools.	CyberRwanda, a digital platform that aims to improve the health and livelihoods of adolescents in Rwanda. The platform allows adolescents to learn integrated skills-building information through edutainment behavior change stories and an extensive frequently asked questions library, order health products online, and link to the program’s network of health care providers who offer adolescent-friendly care.	N/A
Bruns^40^	Men are afraid and don’t feel safe to talk and have no one they trust. They think of HIV treatment and testing in terms of loss and not gain. Negative connotation and experiences in the clinic.	Development of a peer-support intervention called Coach Mpilo that employs men living with HIV who are stable on ART as guides/coaches for men who are newly diagnosed or lost to follow-up.	Preliminary data on the Coach Mpilo intervention showed that more than 90% of men returned to care within the first month of support and remained on treatment after. The intervention reached over 3,700 men living with HIV and employed 120 coaches since March 2020.
Data monitoring and surveillance systems for disease outbreaks			
Durski et al.^42^	Irregular and inaccurate data collection and reporting during the Ebola outbreak.	The Global Ebola Laboratory Data Collection and Reporting System was developed to orient the response at the district, national, and international levels across Guinea, Liberia, and Sierra Leone. This included generating situation reports, monitoring the epidemiological and operational situation, and providing forecasts.	From March 2014 through August 2016, the results of 256,343 specimens were tested for Ebola virus disease in 47 laboratories across the 3 countries. The platform was used to support the 2016 Yellow Fever outbreak in Democratic Republic of Congo and Angola.
Tijani et al.^43^	Lack of prioritization, accountability, and direct consequence for inaccurate data to reduce the misinterpretation of existing data and quality control.	The outbreak management system that was developed had the following features: case reporting form, triaging/eligibility indication, phone number verification, address validation, access control, privacy, contact tracing data, duplicate entry restriction.	The system had processed more than 30,000 reports with more than 75% of these reports considered eligible for testing based on their symptoms. The test center reported it was easier to collect and verify data and report results. (Prototype testing)
Shaikh et al.^44^	Inadequate disease outbreak surveillance. Overburdened clinic staff and overreliance on paper-based log systems.	Tablet-based interface for data entry and a visualization tool for public health officials.	Compliance to the application reached 100% at the end of Day 1. At Day 3 of the Mela, public health officials noticed a spike in diarrheal diseases and dispatched a team of sanitation engineers to test the water supply. The digital disease surveillance has been deployed at 2 of the world’s largest gatherings.
Reframing health service delivery			
Pillsbury et al.^47^	Referral system gaps to improve hypertension control and access to care. Referral barriers (financial, logistical, and infrastructural) and behaviors that influence referral completion.	Development of an implementation strategy that included a peer navigator who assisted with logistical navigation, education, and psychosocial support.	93% of referrals were completed. Participants perceived that the implementation strategy facilitated referral completion. (Prototype testing)
Leung et al.^48^	Access to NCD care while keeping local and economic constraints in mind.	A model for NCD delivery, led by community health educators and rural clinicians, that consisted of microfinance and monthly group medical visits. The intervention included features such as medication availability, financial resources, peer support, and reduced caregiver burden.	Participants reported better access to medical services by mitigating the need to travel and cost of medications. (Prototype testing)
Arrivillaga et al.^49^	Quality and access to cervical cancer prevention.	Encanto, a free service of educational manicure in the health center waiting rooms; No le des la espalda a la citología, a media-based strategy using YouTube, social networks, SMS, and Beacon for women that have never taken a Pap test; Tu Turnero ESE, an educational wireless queuing device to optimize time in the waiting room; Citobot to detect precancerous lesions immediately using artificial intelligence.	N/A
Yadav et al.^50^	Limited health literacy among patients/family and community members. Limited skills and capacity of health workers for delivering COPD care.	A model of integrated care for delivering evidence-based biomedical and psychosocial care to support self-management for people with multimorbid COPD. Features of the model include screening of community members aged older than 40 years with COPD, and management of patients with primary health care.	N/A
van der Westhuizen et al.^51^	Poor adherence to chronic medication in the elderly, as older persons are overwhelmed by changes in their health.	Adherence Hero’s was developed to focus on rewarding residents for adhering to their medication schedules and for providing social support. Another intervention called “Medi-cal” for residents to list their medications and record uptake.	N/A
Ramaswamy et al.^52^	Inadequate essential health resources and support systems in place where PRIME was implemented.	Capacity-building package, implementation support tools, and PRIME service delivery model. They included screening of patients, provision of pharmacological and psychosocial interventions, follow-up of patients, procurement and supply chain management of drugs, and establishment of an information system to monitor progress.	Progression in detection and treatment of patients with depression and alcohol use disorders. 300 and 582 patients were screened for depression and alcohol use in August and December 2014, respectively.
mHealth interventions with end user engagement			
Morse et al.^55^	Challenges with palliative care and pain management for cancer patients in Tanzania.	An intervention, mPCL, included the following features: regular real-time symptom assessment, creating and updating a synoptic clinical record, patient or caregiver and care team communication for care coordination, and symptom-focused educational resources.	N/A
Farao et al.^56^	Poor eyesight. Low literacy and English language proficiency. Under-resourced environment and overburdened healthcare workers.	Redesign of an mHealth app to read the result of the tuberculin skin test for latent TB infection screening.	N/A
Ahonkhai et al.^57^	Lack of ART adherence among young adults.	PEERNaija, a gamified application to support the behavioral change for ART adherence among young adults in Nigeria that aimed to improve self-efficacy and self-regulation, leverage peer relationships to incentivize medication adherence, provide peer social support through app-based chat groups, and allow outreach of the provider team.	N/A
System strengthening in multilayered health sectors			
Andersson et al.^54^	Inadequate use of national reporting tools for service and supply chain data. Poor user experience with the application and logistics data.	Paper-based stock record, cStock smartphone application, community health volunteer supervisor smartphone application, and the unstructured supplementary service data reporting system.	N/A
Ben-Zeev et al.^59^	Lack of awareness about human rights violations and evidence-based mental health care practices among healers.	A smartphone toolkit designed to support the dissemination of evidence-based psychosocial interventions to strengthen human rights awareness among healers.	N/A
Kanagat et al.^60^	Lack of TA programs that focus on capacity building within TA programs, and accountability between and within TA actors.	TA practices toward a more rewarding, effective, and sustainable practice, including related recommended actions and behaviors. Participants suggested governments establishing their own agendas and taking the lead in coordinating TA-based priorities.	N/A
Catalani et al.^61^	Lack of knowledge about isoniazid preventive therapy eligibility, navigating information systems, and lack of access to resources.	They developed detailed treatment protocols, computer-based rules for clinical decision support system, and health communications guidelines for decision support message that providers would receive.	N/A

Abbreviations: ART, antiretroviral therapy; COPD, chronic obstructive pulmonary disorder; N/A, not applicable; NCD, noncommunicable disease; TA, technical assistance.

^a^ Monitoring and evaluation occurred during prototype testing is indicated in parenthesis.

Another important aspect of Phase 3 was rapid testing. A study^39^ that aimed to improve reproductive health among youth in Rwanda tested paper-based prototypes of a digital intervention and gathered rapid feedback on the design, content, and delivery channels. This process allowed participants to share honest feedback, as they might have assumed their feedback would be ineffective if polished prototypes were presented.

Studies that developed digital technology-based prototypes largely relied on quantitative usability testing to measure satisfaction and interface preference. One study^54^ conducted 2 rounds of usability testing surveys for low-fidelity and high-fidelity prototypes of a supply chain. While less common, other sources of data were also collected during testing. A study^56^ from South Africa tested a TB application using user personas (fictitious representations of the intended end users^62^) and conducted observations, think-aloud (verbalizing thoughts while completing a task^63^), and usability assessments among health workers.

Iteration was central to all studies, although its frequency and timeline varied. Most studies conducted 2 cycles of iteration; few studies provided explanations about why they conducted multiple rounds of refinement, where iterations occurred, and when they ceased. It was reported that allowing room for iterations enabled designers and researchers to resolve tensions between designed prototypes and existing evidence.^35^

Iteration was central to all studies although its frequency and timeline varied.

Additionally, some studies^35^^,^^36^^,^^39^^,^^40^^,^^42^^,^^44^^,^^52^^,^^61^ described the full-scale implementation of finalized prototypes leveraging implementing partners and resources. Their experiences with implementation, including insights that informed subsequent iterations, were reported. Some studies conducted additional iterations while others noted the need for further refinement of the interventions. Many of the included studies underscored the importance of exploring how their solutions could be implemented at a larger scale or in larger communities, as well as evaluating the impact.^44^^,^^54^^,^^59^

### Monitoring and Evaluation of Human-Centered Design-Driven Solutions

Of the 8 (36.4%) studies that reported some monitoring and evaluation (M&E) findings of design-based interventions, 5 reported M&E outcomes at the implementation stage after prototype testing (Table 4). For example, a study^40^ from South Africa shared preliminary data on a peer-support intervention that aimed to retain and guide men diagnosed with HIV to complete their treatment, showing that 90% of men who were lost to follow-up returned to care within the first month of support. Three studies reported outcomes of focus (e.g., access to services) at the prototyping phase. However, the health impacts of HCD-derived interventions were seldom measured or reported.

Other articles that did not include information on M&E concluded with descriptions of the interventions designed through HCD and reflections on the process. Some of them pointed to the need for full-scale implementation and evaluation of the solutions while alluding to anticipated challenges, such as lack of funding and complex decision-making across different service units.^43^^,^^49^

### Strengths and Limitations of Human-Centered Design

We identified the following strengths.

- Empathizing with the target population promoted the development of tailored intervention and implementation strategies.
- Health practitioners and implementers were able to make informed decisions based on population needs.
- A sense of ownership among end users and stakeholders was enhanced, improving the effectiveness and sustainability of design-based programs.
- The use of flexible methodologies and tools was encouraged.
- Rapid testing and iterative refinement of prototypes facilitated incremental improvements rather than complete redesigns, reducing costs and resources.
- Stakeholder and leadership buy-in was ensured, which subsequently diversified implementation strategies and scope.

We also identified several limitations.

- Lack of discussion around programmatic accountability and long-term sustainability.
- Challenges in maintaining coordination of multidisciplinary teams.
- Insufficient M&E efforts to assess processes and outcomes of design-based interventions.
- Challenges in sustaining the engagement of people experiencing disadvantages.
- Increased time and resource demands due to multiple cycles of iteration.
- The inherent “ambiguity” mindset of HCD, which can hinder definitive decision-making when developing certain artifacts.

### Key Patterns of Reporting Human-Centered Design

We organized the identified reporting patterns by key design themes.

#### Description of Design Processes

Three articles^35^^,^^42^^,^^48^ enhanced the clarity of how activities occurred within each stage, using HCD-phase subsections in the text. Dissecting phases in both methods and results sections helped the reader understand how certain methods were translated into outcomes.^48^ Despite the innately iterative nature of HCD, it was commonly reported in a linear way, with little indication of whether and how programs had returned to previous design stages. This issue was partly addressed by visual diagrams that displayed the cyclical design process undertaken.^47^^,^^56^

#### Presentation of Data

Findings from end users and stakeholders appeared less structured. Several studies^36^^,^^37^ presented interview excerpts from Phase 1 to depict the challenges faced by end users and the community. Meanwhile, studies presented usability findings from Phase 3 with tables and graphics. A study^59^ showed numeric ratings on the feasibility and acceptability of the intervention among survey respondents. Outputs from various design activities, such as events, flipcharts, or sticky notes, were presented through photographs.^36^^,^^48^^,^^49^

#### Prototype Development and Refinement

Visual aids enhanced the clarity of the prototyping process.^39^^,^^48^ For example, a table displaying how an initial prototype (e.g., village-based health screenings) received user feedback (e.g., concerns about stigmatization) and was subsequently modified (e.g., removing a project logo from vehicles) effectively illustrated this process.^48^ Images of finalized or rough/low-fidelity prototypes were commonly presented to complement the text.^39^^,^^49^^,^^56^^,^^59^

#### Achievement of Design Principles

HCD reporting included explicit descriptions of how projects achieved key design principles, evaluated their approach’s strengths and weaknesses, and outlined implications for future practice. While not common, a study^35^ presented a table that listed 5 core design recommendations (e.g., cultivating empathy) and outlined how their program met these recommended actions by presenting case-based experiences.

HCD reporting included explicit descriptions of how projects achieved key design principles, evaluated their approach’s strengths and weaknesses, and outlined implications for future practice.

#### Stakeholder Engagement

Although the process of stakeholder engagement was commonly provided in the text, visualizing stakeholder mapping (e.g., using a diagram) proved particularly useful, especially when multiple community-based organizations, health institutions, and governmental entities were involved.^42^^,^^48^ A stakeholder mapping helps designers and readers understand the relationships, roles, and level of influence different stakeholders have over a project or design challenge.

#### Tension Management

Many studies ensured transparency by detailing challenges, tensions, and their resolutions. Several studies highlighted issues in sustaining the engagement of young users with high vulnerabilities and obtaining in-depth information from community members. These challenges were addressed through youth-led approaches, staff training, and creative and playful activities to foster participant interest.^35^^,^^36^ Strategies to overcome resource constraints and information about full implementation were less frequently reported.

## DISCUSSION

In this article, we reviewed studies that applied HCD holistically in designing and implementing public health interventions in LMICs. Despite its relatively recent introduction to global health, HCD’s potential appears promising. It affords creative methods to learn from and design with people, thereby making culturally appropriate and acceptable interventions for end users and their community as a whole. HCD is particularly effective in LMICs due to its intentional focus on generating solutions that resonate with local values and goals. Compared with traditional research that relies on a predetermined methodology to assess needs, HCD gives voices to underserved populations and communities throughout the project. Although these tenets are not new to global health practitioners, HCD pushes them to more fully recognize individuals’ identities and experiences and focus on ensuring the desirability and viability of solutions.^26^^,^^64^

The benefits of HCD in engaging and empathizing with end users can be further amplified by the proficiency of design staff, as emphasized by studies focused on designing solutions for highly underresourced groups. Some programs demonstrated the potential of capacity-building among design staff in addressing challenges, such as retaining adolescents or encouraging them to participate openly in group discussions.^35^^,^^36^ Capacity-building of designers, however, was found to be both time- and resource-intensive.^35^ As such, we recognize the need for program implementers and donors to allocate sufficient resources and time to invest in training human-centered designers not only to foster skills but also to strengthen their ethos in working with community resources and making practical decisions amid programmatic and systemic constraints.

Furthermore, our review discovered that HCD optimized stakeholder engagement via both process and research/design, through which a variety of perspectives, knowledge, and experiences were obtained. Leveraging multidisciplinary teams engendered innovative solutions, magnified implementation scope, and strengthened local capacity. Stakeholder engagement ultimately served as sustainable community resources (e.g., support groups) in some studies.^47^^,^^50^

Despite these opportunities, factors that affect the sustainability of solutions must be contemplated. Time and costs required for iterations and consistent commitment from participants and design staff have been cited as major barriers to long-term implementation. When HCD-inspired solutions are sustainable, they can enhance health system performance while also promoting equity and inclusion in health programming in LMICs. A recent study delineated theoretical pathways, illustrating how HCD fosters synergetic relationships between factors and goals within the health sector (e.g., adaptive service delivery) and those in global health ecosystems (e.g., capacity among cross-functional teams).^64^

Our review provided critical insights into how traditional research methods can advance HCD practices. Conducting in-depth interviews or FGDs can be useful for identifying a wide array of challenges/solutions. The effectiveness of these methods is augmented when implemented with an empathetic angle, treating end users and stakeholders as co-creators rather than passive subjects. However, we must acknowledge that HCD’s participatory approach is put to the test when people do not think critically about their needs or the environment they are immersed in. This urges researchers to inquire into local culture, history, and geopolitics, along with a fact-checking procedure to ensure accuracy. Designing data instruments with carefully crafted guiding questions can help individuals critically reflect on their lived experiences and sift through uncharted opportunities.

Our review provided critical insights into how traditional research methods can advance HCD practices.

Advancing research efforts in HCD requires a flexible mindset toward what constitutes “data,” moving beyond the quantitative-qualitative research spectrum. To this end, we recommend leveraging various data sources creatively to uncover critical yet often overlooked aspects of prototypes. For example, providing scenarios from other similar settings and building on familiar objects, concepts, and stories can trigger the idea generation process among participants. Examining local documents (e.g., newspapers and online materials) and institutional records and conducting field research (e.g., market analysis and site visits) will allow researchers to enhance the interpretation and validation of participants’ accounts. While ethnography has a rich history of adopting the methods outlined here, our focus is on ensuring these “ethnographic” approaches are intentionally planned for the design of products, services, or systems from the outset and remain adaptable throughout the design process.

There remains room for improvement across programs that offer digital technology-based solutions. HCD has been predominantly used for digital interventions in high-income countries based on the user-centered approach, where end users often serve as informants rather than co-creators.^4^ We stress the importance of empathizing with user needs at all phases, especially when user requirements conflict with functional requirements or end users have limited digital literacy and familiarity with digital innovations. We found that low-fidelity digital prototypes can boost users’ familiarity with proposed solutions. For example, if participants are resistant to sharing their genuine input on established digital products, the process of drawing and making desired prototypes with people becomes an effective testing methodology. Systematically recording the process and identifying themes that emerge from the procedure would make such data to be used as evidence in conjunction with conventional usability surveys.

Overall, few studies conducted M&E or reported health impacts. The lack of empirical evidence suggests it may have been thwarted by the rigorous standards of traditional global health M&E frameworks, reliant on indicators like coverage, frequency, and uptake. Also, the iterative cycle of refinement, along with the intangibility of design principles, could have prevented proper measurement of HCD processes.^65^ An article suggested 3 approaches to resolving these challenges.^65^ First, HCD’s iterative approach should equally be applied to M&E, allowing evaluations of multiple refinements. Also, measuring proximal indicators (e.g., human desires) of health outcomes and mixing diverse methods should be promoted. Lastly, transparency in documentation should be encouraged among practitioners. A recent study in Mozambique reported how prototype solutions were accepted by end users and resulted in intangible benefits.^66^ M&E frameworks should include indicators that accurately reflect the health issues, target populations, and program logistics in focus, contributing to effective knowledge sharing. We believe that sufficient investment in the creation of M&E frameworks would enhance the rigor of HCD and expand its applicability in science.

Our findings revealed that reporting patterns greatly varied across the literature. Although reports broken down by design phase elucidated how HCD was conducted as an approach, the standard structure of scholarly articles that is usually in a certain sequence somewhat constrained the reporting of how methods and outcomes within a phase gave rise to the next. Overall, visual materials enhanced the clarity of design processes, methods, and outputs across the studies. We suggest the following elements as vital for advancing future reporting: (1) diagrams illustrating a cyclical (rather than linear) HCD approach; (2) multidimensional data presentations using excerpts, graphs, and other formats; (3) visual aids that depict HCD activities and the progression of prototype development; (4) transparency in failures, unforeseen outcomes, and any compromises made; (5) statements about how HCD mindsets and ethics are upheld; and (6) presentation of process and outcome evaluation results.

We must underscore that many studies were excluded from our review, as they did not meet the criteria of conducting or reporting all design phases. While HCD can yield significant impact outside a comprehensive process, we focus on its full potential to optimize benefits. Simply measuring people’s needs does not equate to empathy; continuous reflection on their perspectives throughout intervention development, evaluation, and refinement is what sets HCD apart from other approaches. However, we recognize that adopting an end-to-end approach may require a considerable amount of time. This is especially true because design teams must elicit rich information from participants, synthesize large volumes of data to make targeted design decisions, and conduct multiple iterations of prototype refinements before full implementation. These factors should be carefully considered during program planning to ensure sufficient time allocation and secure the necessary resources.

Properly executed HCD approaches, we believe, can produce outcomes that are more organic to the particular context in which the work is being done, thus facilitating their adoption and scale-up. However, this is an empirical question. We urge future researchers to explicitly test the proposition as to whether adopting an HCD approach is more effective and sustainable in the long run and whether it is also more cost-effective. Overall, with HCD’s recent flourish in LMICs, our study aimed to answer questions about why the approach should be considered in global health, how we can maximize the benefits, and what could be done to improve HCD for global health, noting its limitations to date.

Properly executed HCD approaches, we believe, can produce outcomes that are more organic to the particular context in which the work is being done, thus facilitating their adoption and scale-up.

### Limitations

Our narrative review only included studies that adopted a comprehensive HCD approach and provided substantial details on each phase. This could have eliminated studies that used HCD but did not fully report the process. It is also possible that early studies that applied design principles but did not use the related terminology were also excluded. Since most of the included studies focused on intervention design, our review provided a limited understanding of how HCD interventions were implemented or evaluated. Additional information, such as M&E, may be available elsewhere. We believe that including additional data sources and broadening the inclusion criteria in future research can help address this concern. Lastly, a limitation of this study is the exclusion of gray literature in our review. Including the gray literature would require us to make judgments about quality, potential biases, and institutional credibility that we were not prepared to do. We recommend that future research include gray literature while using strategies to mitigate quality concerns, such as using a quality appraisal tool, focusing on sources with established credibility, and cross-validating findings. Meanwhile, we believe that nudging organizations and funders to promote dissemination through peer-reviewed channels would serve everyone well. Funders should consider properly incentivizing their grantees to publish in peer-reviewed outlets. This may require, among other things, project proposals to explicitly include a timeline in the workplan dedicated to writing peer-reviewed articles.

## RECOMMENDATIONS FOR FUTURE HUMAN-CENTERED DESIGN PRACTICE, RESEARCH, AND REPORTING IN GLOBAL HEALTH PROGRAMS

We list recommendations for practice (how HCD is actually rolled out as part of a project), research (how tools and methods are adapted to the unique ways in which an HCD approach differs from more traditional research approaches), and reporting (the details of the work, including when and how decisions were made). These recommendations are based on effective practices commonly identified across the programs and areas where the programs fell short or lacked proper attention.

### Recommendations for Practice

#### Effective Practices From the Programs

- Strengthen design staff skills to effectively encourage participation among diverse cultural groups, maintain stakeholder engagement, and resolve conflicts in views and priorities.
- Engage in rapid and low-fidelity prototyping to gather quick feedback and minimize costs. Avoid presenting overly polished prototypes to encourage honest feedback from participants.
- Practice key design mindsets throughout HCD practice, including creative confidence, simplicity, learning from failure, empathy, embracing ambiguity, optimism, and an iterative approach.
- Encourage unconventional ideas to creatively address entrenched health problems, recognizing that rural and underresourced communities may be unfamiliar with this approach. Use eye-opening exercises, such as video case studies and games, to broaden community members’ perspectives, especially during the ideation phase.

#### Areas for Growth

- Expand the definition of “user” to include secondary stakeholders, such as family members or local authorities. Ensure all voices are heard, especially those at risk of being overshadowed, by considering literacy levels and group dynamics and engaging stakeholders separately to accurately capture their perspectives.
- Operationalize HCD in an iterative manner by way of responding to insights as they emerge and allowing that to dictate the flow, and ultimately the outcome, of the process.
- Train local designers and workshop facilitators who can speak the local language used by priority groups rather than relying on translators in the process.
- Align HCD processes and solutions with community norms, ensuring this fit via thorough testing. Collaborate with local authorities from the outset to embed solutions within existing systems and support scalability.
- Communicate transparently with stakeholders about how design discussion topics were selected, why certain solution ideas were prioritized, and whether input from external study investigators was incorporated.
- Discuss factors that contribute to the sustainability of HCD solutions early on. Roles and expectations of stakeholders, implementing parties, costs, and logistical issues should be part of the design discussions.

### Recommendations for Research

#### Effective Practices From the Programs

- Leverage both primary and secondary research for empathy by collecting diverse types of data, such as document reviews and field research, in conjunction with direct stakeholder engagement and learning. Data triangulation also contributes to the effectiveness and scientific rigor of an HCD-based intervention.
- Define “data” flexibly in the HCD context. Researchers often need to use creative ways to adapt traditional research methods based on the situation and program context.

#### Areas for Growth

- Develop research tools to properly guide people to critically reflect on their needs and the environment.
- Implement rigorous research methods, particularly ethnographic approaches like participant observation, with a highly trained research team. Engaging and training research personnel affects the timeline and budget, which should be addressed during early design discussions.
- Adopt diverse perspectives beyond those of high-income countries when designing programs offering digital technology solutions, considering end users’ digital literacy, familiarity with technology, and cultural norms.
- Develop M&E frameworks at an early stage to measure both processes (how well the design process is followed) and outcomes (how effective the designed solutions are) unique to a program. Iterative evaluations, assessing intangible outcomes, and data transparency can advance M&E efforts.
- Develop metrics that resonate with the lived experiences of end users. Engage end users in the M&E phase (beyond the design phase) and co-create evaluation tools to ensure that the metrics reflect their priorities and context.
- Include indicators and guiding questions in M&E frameworks to capture the scalability and sustainability of the solutions. This involves testing the solutions with diverse stakeholders and communities and assessing local capacity and available resources.

### Recommendations for Reporting

#### Effective Practices From the Programs

- Report on all design phases to illustrate how HCD practices evolve through stages, ensuring key design principles are embedded in all.
- Visualize activities, processes, and outcomes of a program to enhance understanding, communication, and decision-making among practitioners and donors.
- Provide strategies undertaken for overcoming challenges and tensions throughout the design process to offer practical lessons for future practitioners and decision-makers.

#### Areas for Growth

- Present prototype iterations clearly, ideally with visual aids, to outline decisions on updated elements, unchanged features, and the rationale behind those choices.
- Encourage transparent reporting of failures, unforeseen outcomes, conflicts, and any compromises made, including those that could not be resolved.
- Describe how a program ensures compliance with design principles and mindsets, along with the strategies undertaken to help practitioners adhere to standards and guide future HCD program planning.

## CONCLUSIONS

This review offers learnings on adopting HCD approaches in LMICs, where resource constraints and system gaps are predominant. We encourage design practitioners to engage underserved groups and key stakeholders throughout the design process by pursuing key design mindsets and principles. Data triangulation can be particularly valuable for fostering in-depth empathy with end users, reducing biases in prototype development, and enhancing effectiveness; the definition of “data” is worth surpassing the quantitative-qualitative spectrum in the HCD context. Advancing implementation, evaluation efforts, and reporting practices would enable design practitioners, researchers, and decision-makers to better apply design principles, leading to solutions that are deeply rooted in people’s needs and experiences.

## Notes

**Peer Reviewed**
